# Following the Trace of Cyclodextrins on the Selenium and Tellurium Odyssey

**DOI:** 10.3390/ijms25147799

**Published:** 2024-07-16

**Authors:** Cristina Morán-Serradilla, Daniel Plano, Arun K. Sharma, Carmen Sanmartín

**Affiliations:** 1 Department of Pharmaceutical Sciences, University of Navarra, Irunlarrea 1, E-31008 Pamplona, Spain; cmoran.3@alumni.unav.es (C.M.-S.); dplano@unav.es (D.P.); 2 Department of Pharmacology, Penn State College of Medicine, 500 University Drive, Hershey, PA 17033, USA; asharma1@pennstatehealth.psu.edu; 3 Penn State Cancer Institute, 400 University Drive, Hershey, PA 17033, USA

**Keywords:** drug delivery system, chalcogen compounds, cyclodextrin, inclusion complex, selenium, tellurium

## Abstract

There is an urgent need to develop safer and more effective modalities for the treatment of numerous pathologies due to the increasing rates of drug resistance, undesired side effects, poor clinical outcomes, etc. Over the past decades, cyclodextrins (CDs) have gathered great attention as potential drug carriers due to their ability to enhance their bioactivities and properties. Likewise, selenium (Se) and tellurium (Te) have been extensively studied during the last decades due to their possible therapeutical applications. Although there is limited research on the relationship between Se and Te and CDs, herein, we highlight different representative examples of the advances related to this topic as well as give our view on the future directions of this emerging area of research. This review encompasses three different aspects of this relationship: (1) modification of the structure of the different CDs; (2) formation of host–guest interaction complexes of naïve CDs with Se and Te derivatives in order to overcome specific limitations of the latter; and (3) the use of CDs as catalysts to achieve novel Se and Te compounds.

## 1. Introduction

**Figure 1:**
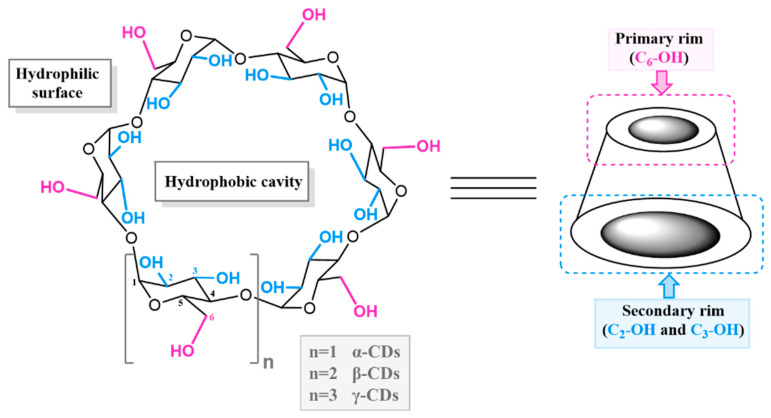
General structure of α-, β-, and γ-CDs.

Chalcogen elements, particularly selenium (Se) and tellurium (Te), have emerged as versatile tools in the design and development of novel bioactive compounds for treating a wide range of pathologies [1,2,3,4,5,6,7,8,9,10,11]. Nevertheless, these compounds usually exhibit poor solubility properties and stability issues under several circumstances (i.e., strong acids or bases and the presence of radicals) that hamper the further exploration of their potential therapeutic applications. The latter are related to the low energy of sigma bonds of C-Se and C-Te and their great nucleophilic features along with their capacity to be attacked by other nucleophiles due to the presence of empty “d” orbitals. To address the poor aqueous solubility and stability issues of Se and Te compounds, cyclodextrins (CDs) and their derivatives have emerged as an effective drug delivery strategy. These macrocyclic glucose oligomers present a toroidal three-dimensional (3D) shape with a hydrophilic outer non-polar inner cavity, the size of which depends on the *D*-glucose units. Amidst them, the most common ones are α-, β-, and γ-CDs, which consist of six, seven, and eight glucopyranose units, respectively (Figure 1). Their architectural features allow them to form guest–host inclusion complexes with a wide variety of molecules such as inorganic, organic, and organometallic compounds. Given these characteristics, they are considered as a promising strategy to ameliorate the bioavailability, solubility, and stability, modulate drug-transport properties, and decrease the release of volatile compounds. Modification at the hydroxyl groups (-OH) is typical of oligosaccharides. The ones present at the C_2_, C_3_, and C_6_ positions in CDs possess nucleophilic properties and can be modified with relative ease. It should be noted that the most basic hydroxyl groups are in position C_6_, whilst the most acidic ones are in C_2_, and the OH groups in C_3_ are the least available ones. Therefore, the primary hydroxyl group (C_6_-OH) will be first attacked by electrophilic reagents. To a certain extent, electrophiles can also attack the secondary hydroxyl groups (2- and 3-position) although they are less favored [12,13,14].

In recent years, the application of CDs as catalysts for organic synthesis has been of intriguing interest as they are a promising alternative toward greener chemical processes. This is due to their easy preparation from available and inexpensive starting materials, eco-friendly nature, facile handling and isolation, non-toxicity, and renewability. The reported protocols for the synthesis of numerous compounds, using CDs as catalysts, are cost-effective, experimentally safe, environmentally friendly, and use water as a solvent [14,15,16,17,18,19,20].

In this context, this review encompasses an overview of the research dedicated towards CDs and Se and Te. The reader is referred to several excellent examples of different chemical modifications of CDs by the hydroxyl groups at the C_2_ and C_6_ positions (Figure 1). Furthermore, we have compiled several research manuscripts about their use as carriers for different selenoderivatives in order to enhance their solubility and stability. In addition, their role in green chemistry will be discussed, as they have been used as catalysts in various reactions to obtain numerous organoselenium compounds. Finally, we will give our opinion about the challenges and future perspectives of this interesting topic.

## 2. Dimeric CDs through a Se or Te Bridge

**Figure 2:**
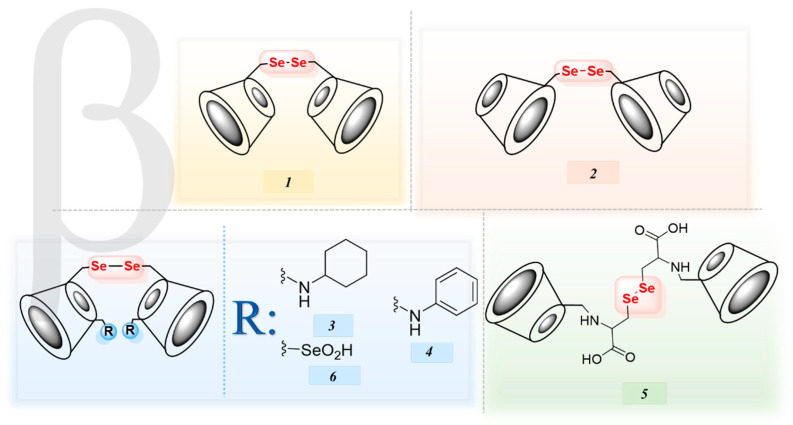
Structure of 6-SeCD (**1**), 2-SeCD (**2**), 6-CySeCD (**3**), 6-AnSeCD (**4**), DisecysCD (**5**), and 6-diSeCD (**6**).

The first Se-bridged CD, diselenide 6,6′-diseleno-bis-β-CD, known as 6-SeCD (**1** in Figure 2), was reported in 1998. It was obtained by attaching a diselenide group to the β-CD primary face, and it exhibited the capacity to reduce H_2_O_2_ by glutathione (GSH). Of note, this activity was 4.3-fold higher than the one reported for ebselen (Table 1). This complex was found to exert its activity through a ping-pong mechanism in its catalytic cycle with a similar kinetic behavior as glutathione peroxidase (GPx). Although its substrate binding ability was not strong, it is believed that the hydrophobic cavity of the CD and the diselenoyl moiety might have played a pivotal role in it [21]. On the other hand, 2,2′-diseleno-bis-β-CD (2-SeCD) (**2** in Figure 2) was reported to exert a rather high GPx activity compared to its analog 6-SeCD and was found to catalyze the reduction of the hydrophobic cumene hydroperoxide (CHP, CuOOH) and *tert*-butyl hydroperoxide (BHP, *t*-buOOH) (Table 1) [22]. This CD-based mimic of GPx also displayed a protective effect on keratinocytes from being injured by ultraviolet B (UV-B) radiation by blocking the generation of reactive oxygen species (ROS) and the UV-B-induced apoptotic signal transduction [23]. Further studies unveiled that it could protect mouse embryonic fibroblast cells (NIHT3) from oxidative stress produced by UV radiation through the regulation of the expression of p53 and Bcl-2, which are key factors for apoptosis. The augmentation of the Bcl-2 protein expression levels by 2-SeCD occurred in a dose-dependent manner, whereas p53 overexpression was inhibited by this Se-bridged CD [24]. Encouraged by these promising results, it has been postulated that 2-SeCD could be a feasible protective skin agent that could overcome some health disorders produced by UV radiation [23].

In the same manner, the potential of 2-SeCD for treating strokes has been evaluated in stroke-prone spontaneously hypertensive rats (SHRsp). In this respect, this complex was found to be able to protect the endothelial cells against oxidative stress by raising the plasma levels of nitric oxide (NO) through the inhibition of hydrogen peroxide generation. Conversely, decreased levels of malondialdehyde (MDA) in plasma and brain tissue were observed in the group treated with high and moderate doses of this Se-bridged CD [25]. In view of the former, it was hypothesized that pretreatment with 2-SeCD could have a protective effect on the liver against hepatic ischemia–reperfusion (I/R), as it has been well documented that the production of ROS and an excessive inflammatory response are some hallmarks linked to this pathology. It was observed that prior treatment with 2-SeCD had an antioxidant activity, as it was able to reduce the levels of MDA as well as hamper the decrease in GSH and myeloperoxidase (MPO) production ascribed to this disorder. Likewise, this complex was able to completely inhibit the generation of MDA in the reperfused rat liver. Keeping in mind the aforesaid antecedents, 2-SeCD can be considered a highly favorable candidate for preventing oxidative stress. Further studies unveiled that 2-SeCD could inhibit I/R-induced apoptosis, as a decrease in the caspase-3 and caspase-9 activity, along with a reduction in DNA fragmentation, was observed. Thereby, it can be concluded that this complex can block the mitochondrial cytochrome c release into the cytosol of the ischemic liver. In order to shed light on its underlying mechanism of action, the effect of 2-SeCD on Bcl-2 family members was studied since they play a pivotal role in the maintenance of the mitochondrial membrane integrity. Data available after the study revealed that the treatment with this complex led to an increased ratio of Bcl-2 to Bax. In view of the aforesaid antecedents, 2-SeCD can be considered a promising candidate for the prevention of the oxidative damage produced in several tissues given its outstanding GPx activity and ameliorated water solubility [26].

A 6A,6A′-dicyclohexylamine-6B,6B′-diselenide-bis-β-CD (6-CySeCD) (**3** in Figure 2) was designed to be a potential GPx mimic and exhibited better activity than 6-SeCD for the reduction of several hydroperoxides (ROOH) such as hydrogen peroxide (H_2_O_2_), CHP, and BHP. It has been hypothesized that the introduction of the amino group near the Se atom might enhance the stability of the nucleophilic intermediate (selenolate), resulting in an enhanced GPx-like activity. Compared with 6-SeCD and ebselen, 6-CySeCD was able to better decrease the mitochondrial swelling produced by oxidative damage. In the same way, a significant decrease in thiobarbituric acid reactive substances (TBARSs), used as markers for lipid peroxidation and seen during mitochondrial damage, was observed in a dose-dependent manner [27]. Of note, the antioxidative effects of this complex were also assessed in cultured immortalized human keratinocyte cells (HaCaT) damaged by UV-B irradiation. According to the cell viability results, 6-CySeCD and ebselen were cytotoxic to these cells at concentrations higher than 1000 µmol/L and 40 µmol/L, respectively. Moreover, this Se-bridged CD was able to effectively inhibit lipid peroxidation and exerted a protective activity that could be related to its capacity to block the generation of ROS. The data obtained from this study indicate that 6-CySeCD was able to inhibit UV-B-induced apoptosis, which was accompanied by a rise in DNA synthesis and cell proliferation, as well as influence the cell cycle distribution of the HaCaT cells. It should also be noted that pretreatment with 6-CySeCD in these cells presented greater protective effects than those obtained with ebselen [28]. In this line, 6A,6A′-dianiline-6B,6B′-diselenide-bis-β-CD (6-AnSeCD, **4** in Figure 2), with an aniline group incorporated in the vicinity of the Se atom, showed a great GPx activity but lower than that of 6-CySeCD. This might be related to the flexibility of the cyclohexylamine group that could facilitate the introduction of the substrate into the cavity of the CD [29].

**Table 1:** The GPx activity of several selenobridged β-CDs, ebselen, PhSeSePh, SeCys, β-CD itself, and the combination of β-CD with SeCys [21,22,26,27,30,31].

Compound	Hydroperoxide	Activity (U/µmol)
**1**	H_2_O_2_	4.2
**2**	H_2_O_2_	7.4
	BHP	4.0
	CHP	10.5
**5**	H_2_O_2_	4.13
	BHP	2.11
	CHP	5.82
**6**	H_2_O_2_	13.5
Ebselen	H_2_O_2_	0.99
SeCys	H_2_O_2_	0.05
PhSeSePh	H_2_O_2_	1.95
β-CD	H_2_O_2_	0.03
β-CD + SeCys	H_2_O_2_	0.11

The binding of selenocysteine (SeCys) to the primary side of β-CD rendered a Se-bridged 6,6′-amino-SeCys-6,6′-deoxy-di-β-CD, known as DisecysCD (**5** in Figure 2). The results available after the study of its GPx (Table 1) indicate that this complex was able to reduce H_2_O_2_ by GSH better than ebselen, SeCys, and diphenyl diselenide (PhSeSePh). It is noteworthy to mention that the activity of β-CD itself and its combination with SeCys was also evaluated, as this amino acid is the catalytically active group of this enzyme. The remarkable acceleration of the reaction between GSH and H_2_O_2_ by this Se-bridged CD compared to the other compounds assessed in the study could be related to an improved interaction between this complex and GSH along with the important role of the hydrophobic cavity of β-CD. Furthermore, DisecysCD was able to catalyze the reduction of BHP and CHP. Notably, the GPx activity for this second substrate was almost 3-fold higher than that for the first one. The likely explanation of these results may be related to the fact that CDs have been reported to bind more robustly in the cavity to shape-/size-fit molecules, such as the aromatic group present in the organic CHP, than shape-/size-unfit compounds [30].

A 6A,6B-diseleninic acid 6A′,6B′-Se-bridged β-CD, 6-diSeCD (**6** in Figure 2), has also been demonstrated to be an excellent GPx mimic (Table 1) and to exert a dose-dependent protective effect in a model of ferrous sulfate-/ascorbate-induced mitochondrial damage due to its antioxidant activity [31].

**Figure 3:**
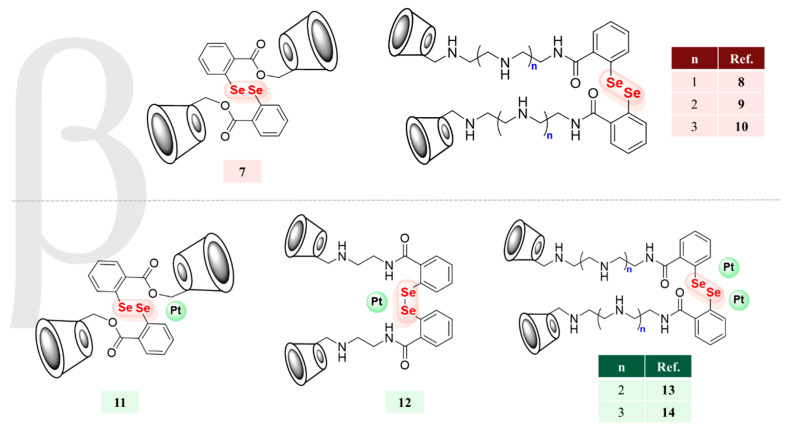
Structure of the bis-β-CDs linked by a 2,2′-diselenobis(benzoyl) spacer along with its corresponding platinum (Pt) (IV) complexes herein reported.

A library of novel bridged bis-β-CDs with a 2,2′-diselenobis(benzoyl) linkage connected with a variety of oligo(ethylenediamine) (**7**–**10**) as well as their corresponding platinum (Pt) (IV) complexes (**11**–**14**) has also been reported (Figure 3). The experimental results obtained showed that these dimers coordinate with one or two ions to render 1:1 or 1:2 metallobridged bis-β-CDs which can further switch the original molecular binding behavior by this coordinated metal to orientate two β-CD cavities to fit the shape of the guest compound. Pt (IV) can also act as a supplemental site of guest recognition by an electrostatic and/or coordinated interaction. The complexation thermodynamics of **7**–**10** with selected substrates, such as the fluorescent dyes 8-anilinonaphthalene-1-sulfonic acid (ANS) and 6-*p*-toluidinylnaphthalene-2-sulfonate (TNS), were also studied. From the data, it seems clear that this inclusion complexation is primarily enthalpy-driven. Additionally, both Van der Waals and hydrophobic interactions were proven to be involved in the stability of the host–guest inclusion complex [32].

**Figure 4:**
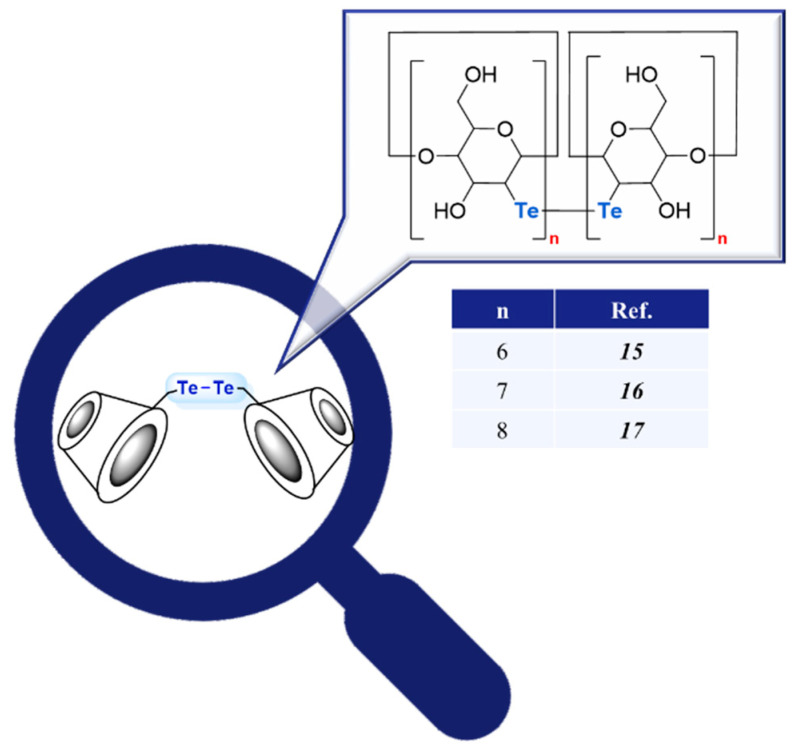
Schematic illustration of the chemical structures of 2-TeCD (**15**), 2-α-TeCD (**16**), and 2-γ-TeCD (**17**).

A 2,2′-Te-bridged β-CD (2-TeCD), **15** in Figure 4, was found to exert a GPx-like activity 46 times greater than ebselen (Table 2). This potent behavior allowed the compound to effectively scavenge hydroperoxides, thus protecting the mitochondria from oxidative damage. Furthermore, it was observed that the abnormal swelling of this organelle was decreased in a dose-dependent way, as was the amount of accumulated MDA [33]. Another study corroborated that an efficient substrate binding is required for the catalytic activity of 2-TeCD. This GPx mimic can effectively and selectively reduce the ROOH by 4-nitrothiophenol (NBSH), a thiol substrate. This decrease was about 3.4 × 10^5^ times greater than the one reported for PhSeSePh. It was proven that hydrophobic interactions along with steric and hydrogen-bonding interactions play a key role during the molecular recognition of this Te-bearing compound [34]. Another article reported the increased substrate specificity and catalytic potency when 5-mercapto-2-nitrobenzoic acid (ArSH) was used as a preferential thiol substrate. These properties, along with water solubility and thermal stability, suggest that 2-TeCD could be a feasible alternative for the study of enzymatic specificity and further potential therapeutic applications [35]. On the other hand, this GPx mimic was assessed to determine its effect on the tumor necrosis factor-α (TNF-α) expression of adhesion molecules (intercellular adhesion molecules [ICAM-1] and vascular cell adhesion molecules [VCAM-1]) and monocyte adhesion, which are involved in the pathogenesis of atherogenesis in human umbilical vein endothelial cells (HUVECs). It was found that 2-TeCD was able to effectively suppress the expression of these molecules on HUVECs’ surface in a concentration-related way as well as inhibit THP-1 monocyte cell adherence to these cells [36].

Another study reported the formation of the stable complexes 2-TeCD and 2-SeCD with an *S*-substituted dinitrophenyl glutathione. This molecule was used instead of GSH, as its binding properties can be more easily monitored by spectroscopic methods. Both CDs were demonstrated to ameliorate the substrate binding property of the S-substituted dinitrophenyl glutathione. However, the activity of 2-TeCD was more pronounced than that of Se-bridged β-CD. It is tempting to speculate that this bridged linkage can provide a more appropriate length and rigidity for the *S*-substituted dinitrophenyl glutathione. In fact, Te presents a larger radius and reduced electronegativity compared with the Se atom and can form more flexible -Te-Te- bonds. Therefore, it can be concluded that this linkage can be a versatile coordinating site that can have an influence on the orientation and binding properties of the CD dimers. Furthermore, a stronger hydrophobic interaction and solvent reorganization was observed in 2-TeCD and 2-SeCD complexes compared to the unmodified β-CD [37].

Further studies assessed whether the use of α- and γ-CDs could have an impact when forming the inclusion complexes and the GPx activity, as they present smaller and larger cavities, respectively. Therefore, 2,2′-Te-bridged α-CD (2-α-TeCD) and 2,2′-Te-bridged γ-CD (2-γ-TeCD) dimers were synthesized (**16** and **17** in Figure 4, respectively). According to the data obtained from the study of their GPx-mimicking activities, a likely linkage between the increasing size of their cavity and their ameliorated catalytic efficiency can be observed. The GPx-mimetic activity of 2-α-TeCD and 2-γ-TeCD was found to be substrate-specific, CHP being the preferred one [38].

**Table 2:** GPx-like activity against H_2_O_2_, BHP, and CHP of ebselen and several Te-bridged CDs [33,38].

	GPx-Mimicking Activities (U/µmol)		
Ref.	H_2_O_2_	BHP	CHP
Ebselen	0.99 ± 0.01	0.33 ± 0.01	1.26 ± 0.01
**15**	34.4 ± 0.17	23.8 ± 0.10	-
**16**	46.7 ± 0.20	32.3 ± 0.15	87.3 ± 0.26
**17**	80.5 ± 0.36	109.8 ± 0.38	149.6 ± 0.43

**Figure 5:**
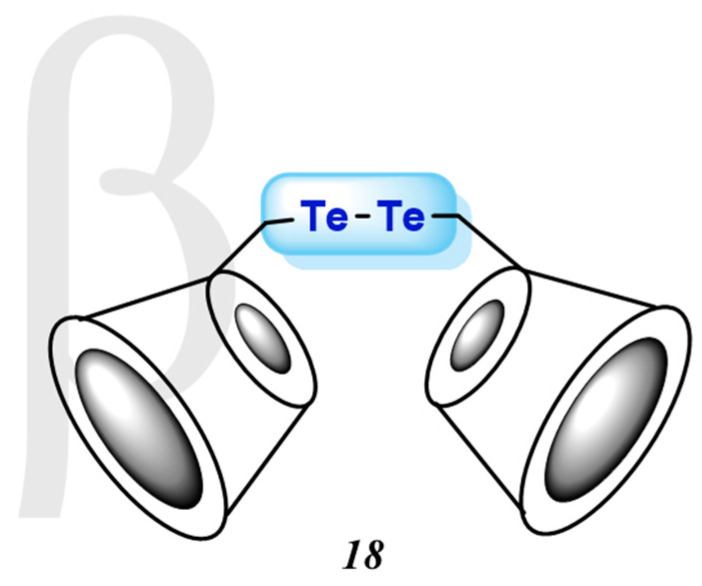
Structure of 6-TeCD (**18**).

The thiol peroxidase activity of the GPx mimic 6,6′-ditellurobis(6-deoxy-β-CD), known as 6-TeCD (Figure 5), has also been reported. This complex showed a strong substrate specificity for ArSH rather than GSH. Additionally, the results available after the study suggested that its catalytic activity depends on the nature of the hydroperoxides. It was found that this complex was able to reduce CHP and BHP at higher rates than the hydrophilic H_2_O_2_, since it was not able to effectively bind into the hydrophobic cavity of β-CD. According to the data depicted in Table 3, the thiol peroxidase activity exerted by this GPx mimic was almost 10^5^-fold greater than the one reported for PhSeSePh and diphenyl ditelluride (PhTeTePh). Of note, these two compounds do not carry any binding group for substrates compared to this Te-bridged CD [39].

**Figure 6:**
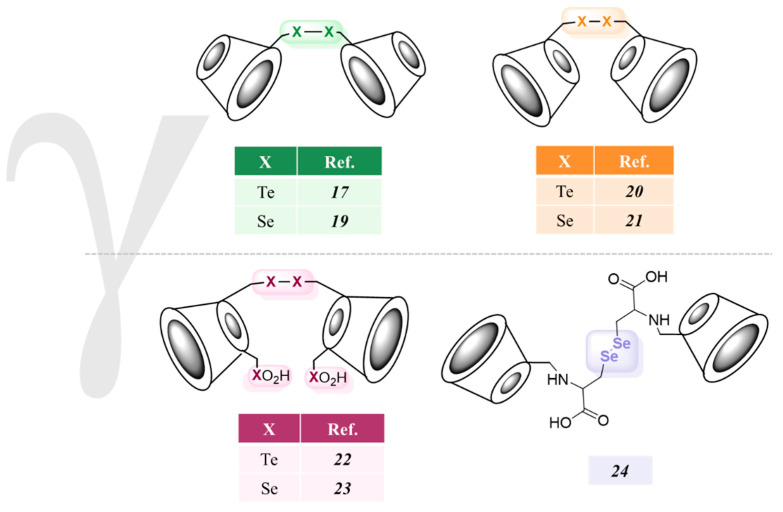
Schematic illustration of the chemical structures of 2-γ-TeCD (**17**), 2-γ-SeCD (**19**), 6-Te-γ-CD (**20**), 6-Se-γ-CD (**21**), 6-diTe-γ-CD (**22**), 6-diSe-γ-CD (**23**), and Disecys-γ-CD (**24**).

On the other hand, the γ-CD scaffold was used as the enzyme model to render a variety of organochalcogen-bridged CD dimers (Figure 6) [40].

Several conclusions could be drawn after the study of these compounds. Firstly, they exhibited better activity than ebselen, and, amidst them, **22** displayed the most efficient peroxidase activity, reducing CHP almost 670-fold more than this experimental drug. Notably, Te compounds showed greater catalytic potency compared to their Se analogues. Another fact that should be remarked is that the modification of the 2-position of the γ-CDs was found to be more efficient than that of the 6-position in most of the cases (Table 4). According to the data gathered in Table 4, it can be observed that CHP was reduced faster than the more hydrophilic BHP and H_2_O_2_. Among all the tested compounds, **22** stood out as the most potent compound. The likely explanation behind this relatively high GPx activity is that the bulky group of CHP can strongly bind into this Te-CD dimer. In addition, compounds **17** and **22** were demonstrated to interfere with the mitochondrial system and reduce oxidative stress, thus protecting the mitochondria against ferrous sulfate-/ascorbate-induced oxidative damage. In view of the former, both Te-bridged CDs can be considered an attractive potential drug alternative for the treatment of ROS-mediated diseases [40].

## 3. CDs Monosubstituted with Organochalcogen Compounds at Position 6

Increasing knowledge on supramolecular chemistry has equipped the scientific community with a toolbox to achieve breakthroughs in the discovery of innovative and potential therapeutic agents. There is a significant body of work devoted to the use of this chemistry in order to attain the synthesis of macromolecules that can mimic the action of certain enzymes. In this regard, great attention has been devoted to CDs, as they exhibit great water solubility, and their unique structure allows them to form host–guest interactions with a great variety of molecules [41,42,43,44].

**Figure 7:**
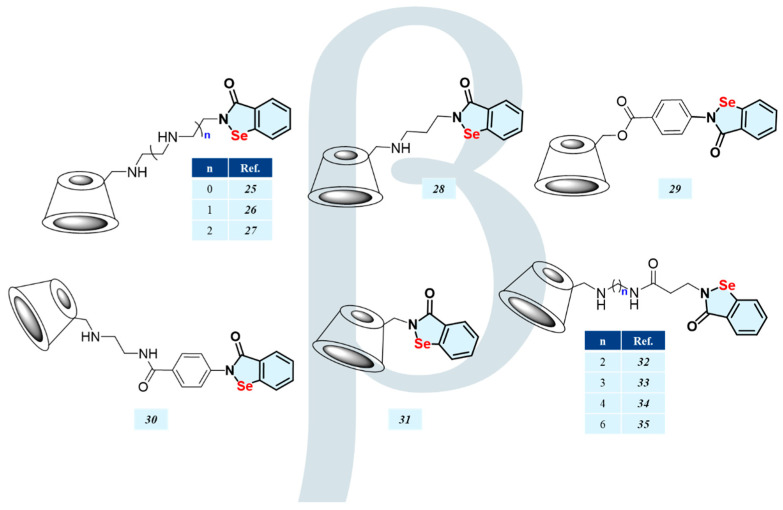
Chemical structure of the described β-CDs with the functional moiety of ebselen.

A library of bifunctional enzyme models (**25**–**30** in Figure 7) was synthesized by the incorporation of a 1,2-benzisoselenazol-3(2*H*)-one moiety at the primary rim of β-CD. The prime aim of the study was to obtain molecular mimics of superoxide dismutase (SOD) and GPx, as these enzymes play an important role in the antioxidative defense system against ROS and free radicals that are responsible of many pathologies [45].

Regarding their mimicking results, it can be observed that the β-CD cavity is only responsible for binding the substrate, as neither the native nor the oligoamino-modified β-CDs (β-CDen, β-CDdien, β-CDtrien, and β-CDprn) had any effect on SOD. On the contrary, all the selenoderivatives exhibited SOD activity, compound **26** being the most active. This might be attributed to the suitable flexibility and length of its oligoamino chain that can lead to a better cooperation relationship between the substrate binding site and the catalytic group (the 1,2-benzisoselenazol-3(2*H*)-one moiety). The lower activity observed for compound **29** could be due to its self-included moiety which impedes the substrate’s inclusion. Surprisingly, compound **30**, which presents the catalytic group outside the β-CD cavity, also displayed a relatively low efficacy towards this enzyme. This could be related to the rigidity of the linker between the oligoamino chain and 1,2-benzisoselenazol-3(2*H*)-one moiety, thus hindering the approach of the catalytic group to the β-CD cavity. The SOD activity results are summarized in Table 5 [45].

Despite the fact that none of the first selenoderivatives (**25**–**28**) synthesized exerted better GPx-mimetic activities than ebselen (Table 6), they were demonstrated to present greater water solubility. Therefore, it was hypothesized that the 2-phenyl group present in ebselen might be pivotal for its GPx activity. Even though compound **29** was expected to be more effective than the other derivatives, as it presents the whole structure of the unmodified parent compound, it exhibited a weak activity. This might be ascribed to the self-inclusion of its modifying moiety that could be responsible for its low water solubility. In view of the former, mimic **30** was synthesized in order to surmount these limitations. In fact, it displayed the best GPx-mimicking activity of all the Se-bearing compounds (0.86 U/µmol) and presented good solubility in water [45].

Likewise, the introduction of the functional moiety of ebselen, the benzoisoselenazolone group, in the structure of β-CD resulted in a series of five novel cyclomaltoheptaose compounds (**31**–**35** in Figure 7) with improved water solubility and a flexible linkage between the catalytic group and the CD complex [46].

**Figure 8:**
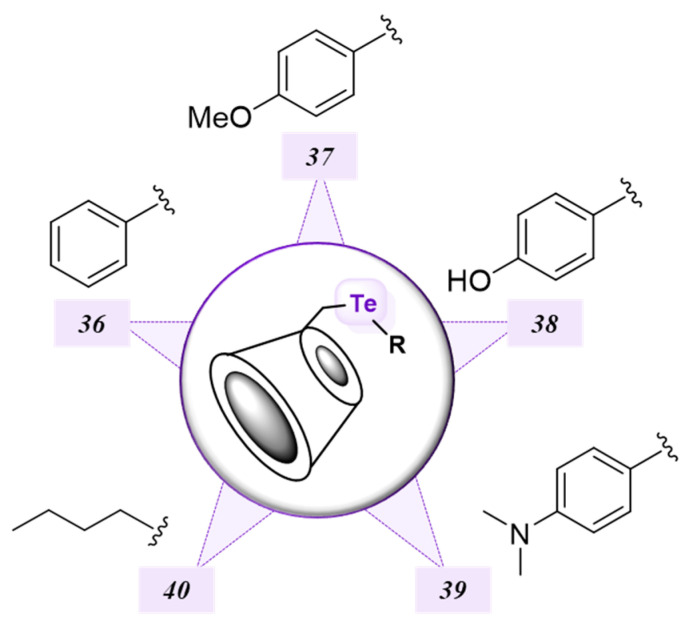
Chemical structure of the β-CD-derived organotellurium compounds.

Another study reported the synthesis of several Te-based CDs (**36**–**40** in Figure 8) and 6-SeCD that could act as GPx mimics [47].

Their antioxidant activities were assessed in order to determine their ability to catalyze the reduction of H_2_O_2_, CHP, and BHP in the presence of GSH, nicotinamide adenine dinucleotide phosphate (NADPH), and GSH reductase (Table 7). In this respect, 6-SeCD did not exert any activity against the tested hydroperoxides. Among the organotellurium derivatives, **40**, which presents an *n*-butyltelluro group, exhibited the best peroxide decomposing activity. This result corroborates previously reported findings that indicate that alkyltelluro groups render better catalysts than the aryltelluro moieties [47,48]. Of note, some of these compounds (**38**–**40**) with good catalytic activity also presented a good combined thioredoxin (Trx) and Trx reductase (TrxR) inhibitory effect (Table 7). Nonetheless, a decreased inhibition efficacy was observed for **36** and **40** when only TrxR was assessed. In the same work, the cytotoxic effect of these compounds was evaluated on a breast cancer cell line (MCF-7), as these cells are characterized by an elevated Trx gene expression. The 4-hydroxyphenyltelluro-derived CD (**38**) exerted an outstanding anticancer activity, whereas the other compounds only displayed a moderate effect, with half-maximal inhibitory concentration (IC_50_) values ranging from 4.6 to 10.4 µM (Table 7) [47].

## 4. CDs Containing Selenocompounds as Guests

Despite the promising properties of CDs, little attention has hitherto been paid to exploring the therapeutic potential of formulating them with different bioactive selenocompounds. A large body of existing literature indicates that synthetic organic selenocompounds are usually poorly soluble in water which hampers their further clinical exploitability, as it prevents them from achieving therapeutically significant doses in blood [49]. Therefore, the use of CDs as carriers for these Se-bearing molecules could bring a new horizon for the development of more soluble and stable compounds. Herein, we highlight representative examples of this strategy that could generate major steps forward in the drug discovery field.

**Figure 9:**
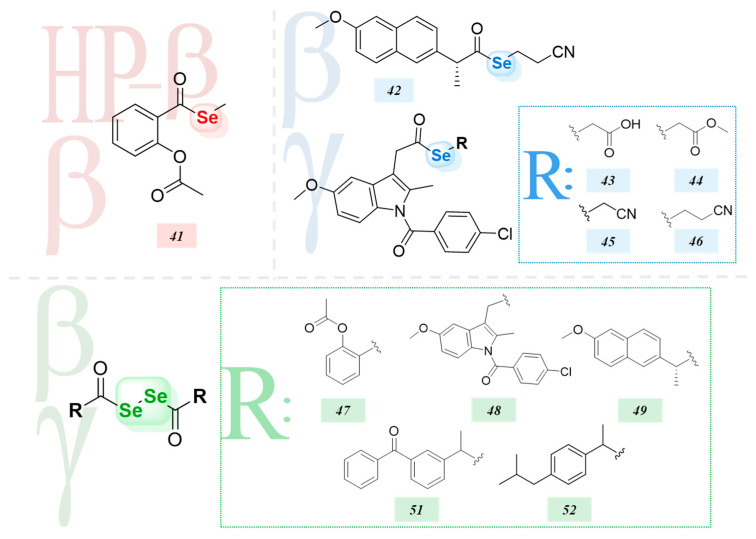
Schematic representation of the β-, HP-β-, and γ-CDs containing selenocompounds as guests.

A novel methylseleno-aspirin (ASA) analog (**41** in Figure 9) was proven to have an outstanding antitumoral activity with a mean cell growth value of 54.63% toward the cancer cell lines of the National Cancer Institute (NCI)-60 cell line screening panel. However, this compound exhibited some drawbacks such as its poor water solubility and its volatile nature which resulted in a decrease in its antitumoral activity. In order to surmount these liabilities and improve its bioavailability, it was formulated with α-, β-, 2-hydroxypropyl-β-, and γ-CDs. Amidst all of them, only β- and 2-hydroxypropyl-β-CDs improved the solubility of this compound. As **41**, dissolved in dimethyl sulfoxide (DMSO), had shown potent activity against colorectal cancer (CRC) cells, these complexes and the non-formulated parent compound were evaluated toward several CRC cell lines (HT-29, HCT-116, RKO, and Caco-2). Of note, **41** exhibited an increased antitumoral efficacy against RKO cells when forming the CD inclusion even at 24 h. Conversely, for the rest of the assessed cell lines, its cytotoxic potential was reduced at that time, although the antitumoral activity was recovered within 72 h. The likely explanation for this finding is that CDs endowed this Se-ASA analog with a more sustained release of the parent compound or its active metabolites (such as methylselenol) over time. Another fact that should be pointed out is that the complex between **41** and β-CD exerted a greater effect than the one with 2-hydroxypropyl-β-CD, which agrees with the greater affinity constant obtained for this latter CD [50]. In view of the former, it can be suggested that the vehiculation of selenocompounds with solubility issues and/or a volatile nature could be a feasible approach to improve their pharmacological potential and continue the drug discovery process.

The improvement in the solubility of a series of Se-non-steroidal anti-inflammatory drug (NSAID) analogs (**42**–**51** in Figure 9) by formulating them with β- and γ-CDs has recently been reported (Table 8) [51]. Amidst all of them, **42** and **51** stood out due to the striking enhancement in their water solubility after forming the inclusion complexes with these CDs. In fact, **51**, one of the diacyl diselenide derivatives with proven outstanding anticancer properties [52], was the most soluble one with an increase of 250- and 350-fold with β- and γ-CD, respectively [51].

Of note, an in silico study was carried out to corroborate these experimental data and determine the possible mechanism of complexation between the organoselenium compounds and the corresponding CDs. Regarding the β-CD-based derivatives, it was found that **42** and **51** exhibited the best results in terms of theoretical complex stability and score. It should also be remarked that these compounds were the ones able to form the most stable and effective guest–host inclusion complexes with γ-CDs, albeit these CDs were expected to form significant interactions with all the Se derivatives studied since they are characterized by a larger inner cavity than β-CDs. Keeping in mind the aforementioned preliminary study, CD-based complexes can be a feasible strategy to improve the bioavailability and solubility of a wide range of interesting Se-containing compounds [51].

Envisioning the need for novel agents for the treatment and prevention of human immune deficiency virus (HIV) infections, a rapidly soluble film of ebselen, a selenocompound with proven anti-HIV and antifungal activities [53,54,55,56,57,58,59], was synthesized with the dual purpose of treating vulvovaginal candidiasis (VVC) and being a pre-exposure prophylactic (PrEP) formulation toward these viruses. Therefore, ebselen, β-CD, and Soluplus^®^ were formulated in a ratio of 1:10:10 as a ternary complex (EβpolySol). It should be noted that the β-CD polymer was selected among others (2-hydroxypropyl-β-CD and sulfobutylether β-CD), as it exhibited an ameliorated water solubility and decreased cytotoxicity of the free drug on HeLa cells. Furthermore, it was found that the inclusion of this selenocompound into the cavity of this CD protected it from thermal degradation. Another fact that should be highlighted is that Soluplus^®^ was added into the formulation to decrease the number of CD polymers required to solubilize ebselen, increase its hydrophilicity, and keep the supersaturation of the parent compound when it is present in the body fluid. In fact, both the micelles of this polymeric stabilizer and the β-CD polymer were found to impede the precipitation of ebselen in the aqueous phase and help to keep the integrity of the inclusion complex once it was dispersed in the polymeric matrix. Additionally, a topical PrEP formulation was obtained by incorporating EβpolySol into a polymeric matrix of polyvinyl alcohol (PVA) and glycerol (20%/2%) to form a film in order to increase its in vivo solubilization and retention time in the vagina and ameliorate its long-term stability. Regarding the safety of this film to the cervical cells, it was hypothesized that it could further counter the infection, ameliorate the symptoms ascribed to these pathologies, and avert further impairment in the mucosal cells. Conversely to the non-formulated parent compound, the EβpolySol film was rapidly and completely disintegrated within 30 s and exhibited a full drug release from the polymeric matrix in almost 30 min according to several in vitro assays. Also, no precipitation of ebselen was found; thereby, all the drug is solubilized in the vaginal fluid. Another fact that should be noted is that this Se-bearing molecule was non-toxic to the tight junctions, which play an important role in protecting the cells from viruses. Thus, the risk of HIV transmission is diminished compared to other marketed vaginal films such as VCF^®^ [60].

Regarding its in vitro antifungal evaluation, *Candida albicans* and *Candida glabrata* were the two strains studied, as they are the main virulent pathogens involved in VVC. From Table 9, it can be observed that both free ebselen and the EβpolySol film exhibited the same minimum inhibitory concentration (MIC) values in both strains, which were superior to the ones obtained for fluconazole and miconazole. Nevertheless, considering that the total amount of this organoselenium compound loaded into the film was almost 125-fold higher than the intended MIC and regarding its proven ameliorated safety to cervical cells, it can be considered a better candidate to decrease the vaginal fungal burden [60].

On the other hand, the anti-HIV-1 activity of ebselen and the film formulation was assessed in several cell lines, and a similar potency for both was observed, as they exhibited IC_50_ values of 0.71 and 0.59 µM, respectively. Noteworthily, the EβpolySol film displayed lower toxicity toward the HeLa-CD4-LTR-LacZ cell line compared to the parent compound. Furthermore, it was found to be non-toxic to the Jurkat cells, which are T lymphocytes, as well as able to decrease the levels of HIV mRNA expressed in these cells. Regarding its adequate mechanical properties and potent anti-HIV and antifungal activities, it is tempting to speculate that this formulation can be considered a feasible therapeutic option for the PrEP treatment of these infections and circumvent some of the risk factors ascribed to them such as VVC [60].

## 5. CDs as Catalysts for Obtaining Selenocompounds

It is a well-known fact that an ample number of drawbacks (e.g., sustainability shortfalls, low solubility, side effects, and environmental hazards) associated with numerous compounds have hampered their further development as commercial therapeutic agents. Green chemistry is gradually emerging in the scientific community, as the use of sustainable chemicals and solvents offers better prospects for development in the pharmaceutical industry [61,62,63]. In this regard, CDs have arisen as a feasible strategy toward the greener chemical synthesis of novel bioactive agents, and their use as catalysts that operate in aqueous solutions has gathered great interest in the past years [64,65,66,67,68]. In this review, we present an overview of the most representative examples of the synthesis of numerous organoselenium compounds catalyzed by CDs in water.

**Scheme 1:**
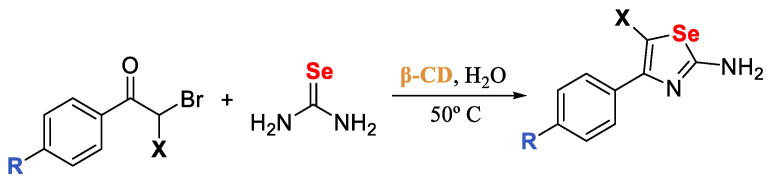
Synthesis of selenazoles (**52**–**61**) using α-bromo ketones, β-CD as catalyst, and selenoureas in water [73].

Even if selenazoles showcase several striking biological activities [69,70,71,72], their further drug development is usually hindered by several shortcomings. Focusing on the synthetic routes reported for these compounds, there are several points of interest such as the broad range of liabilities (e.g., the use of inert atmosphere, anhydrous solvents, long-term reaction, poor yields, and basic conditions) associated with them. Also, the selenoureas used in these reactions are light- and air-sensitive. Encouraged by the need to develop a safer and more efficient, economical, and eco-friendly synthetic methodology to render these interesting compounds, the synthesis of several selenazoles using α-bromo ketones, CD complexes, and selenoureas in water was carried out (Scheme 1) [73].

It should be highlighted that the reactions did not take place with α-CDs, and therefore, β-CDs were chosen as catalysts. Also, the latter are easily accessible and can be recovered and reused. It is worth mentioning that almost all the proposed compounds were obtained in high quantitative yields (86–95%), and no byproducts were detected from the reactions. From the data (Table 10), it seems clear that the use of aromatic α-bromo ketones (**52**–**59**) led to higher yields compared to the aliphatic ones (**60** and **61**). In the same work, they evaluated the substantial role of β-CDs as promoters of the reaction. It was observed that their absence resulted in water solubility problems for some of the compounds, longer reaction times (>12 h), and sparse yields [73].

**Scheme 2:**
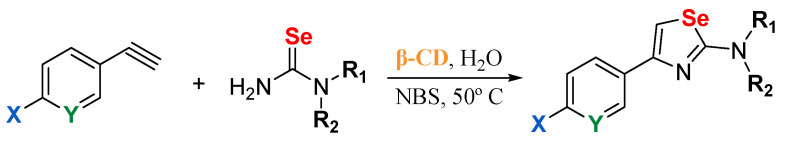
One-pot tandem synthetic strategy for obtaining selenazoles (**62**–**66**) using β-CDs as catalysts in water [74].

**Scheme 3:**
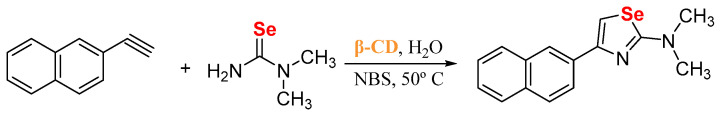
Synthetic procedure to obtain compound **67** [74].

In continuation with the efforts devoted to the development of greener methodologies for the synthesis of selenazole derivatives in aqueous phases, the first one-pot tandem synthetic strategy was reported in 2012. Compounds **62**–**66** were synthesized as summarized in Scheme 2, whereas **67** was obtained as reported in Scheme 3 [74].

The reaction between different selenoureas and phenylacetylenes with *N*-bromo succinimide (NBS) mediated by β-CD was carried out in water and resulted in encouraging yields. It was observed that the use of NBS was crucial, as the reaction did not proceed without it, whereas the absence of the CD led to lower yields of the desired compounds. Remarkably, the substituents of the different selenoureas and phenylacetylenes did not have a significant effect on the efficacy of the reaction (Table 11) [74].

**Scheme 4:**
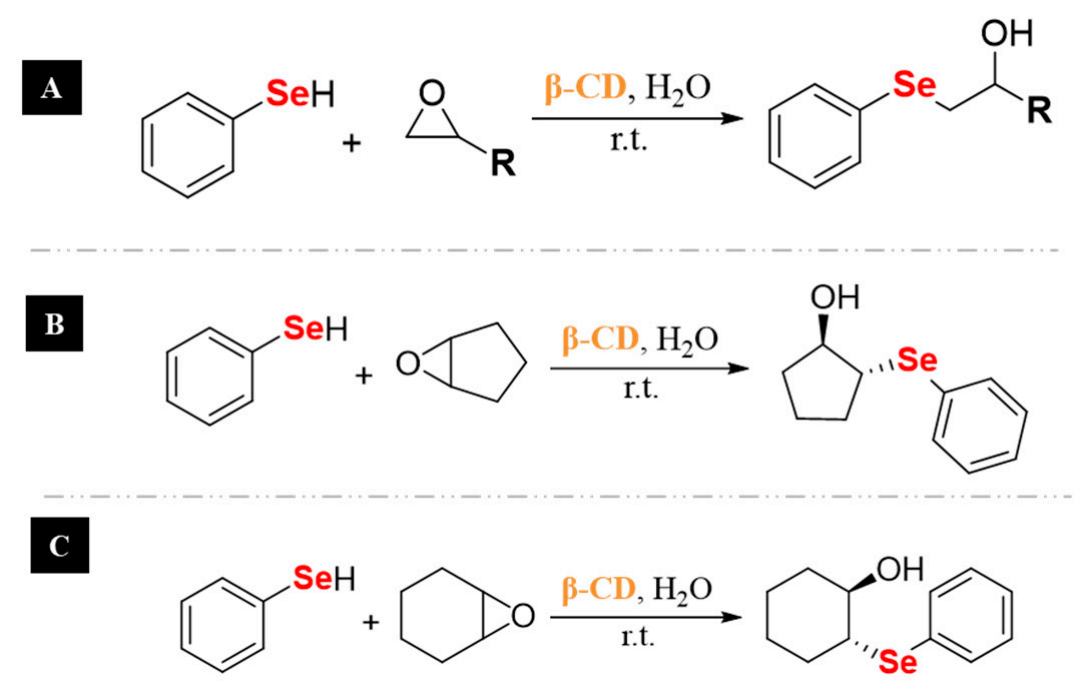
(**A**) Synthesis of β-hydroxy selenides (**68**–**75**) by the regioselective ring opening of oxiranes with benzeneselenol catalyzed by β-CDs in water. (**B**,**C**) Specific synthetic procedures for obtaining compounds **76** and **77**, respectively [75].

On the other hand, the regioselective ring opening of oxiranes with benzeneselenol catalyzed by β-CDs in water rendered a series of β-hydroxy selenides (Scheme 4) with great yields (75–86%) (Table 12). The reactions could also proceed with α-CDs in the same way, albeit β-CDs were chosen, as they are more economical and reusable and can be readily accessible [75].

**Scheme 5:**
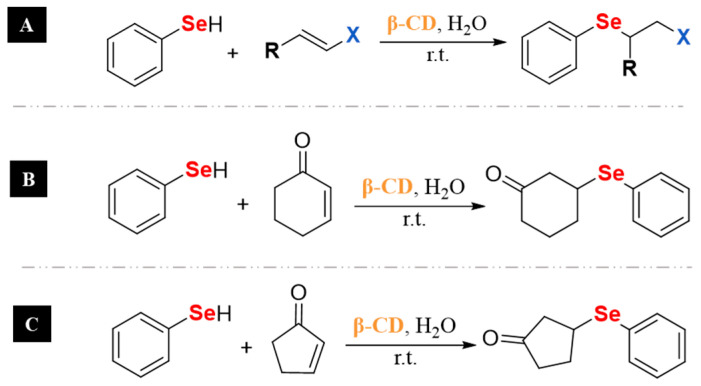
(**A**) Synthesis of β-(phenylseleno)-substituted derivatives (**78**–**85**) by the Michael addition reaction of conjugated alkenes with benzeneselenol catalyzed by β-CDs in water. (**B**,**C**) Specific synthetic procedures for obtaining compounds **86** and **87**, respectively [76].

Likewise, another study unveiled an innovative procedure for the synthesis of β-(phenylseleno)-substituted derivatives through the Michael addition reaction of a great variety of conjugated alkenes with benzeneselenol in the presence of β-CD in water, as reported in Scheme 5. This catalyst plays a pivotal role in the reaction as it activates the benzeneselenol as well as promotes the conjugate addition to the olefins by the formation of an inclusion complex. The results reported indicated that the reaction was highly effective, with yields in the range of 80–88%, although the required reaction time was 20–45 h depending on the initial substrates (Table 13) [76].

**Scheme 6:**
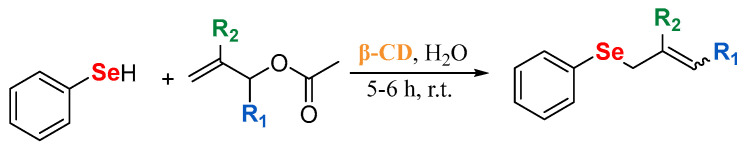
Synthetic procedure for obtaining stereoselective allyl aryl selenides (**88**–**94**) from several Baylis–Hillman acetates and benzeneselenol catalyzed by β-CD in water [77].

Furthermore, a novel protocol has been reported for obtaining stereoselective (*Z*)- and (*E*)-allyl aryl selenides (Scheme 6) from several *Baylis–Hillman* acetates, which are insoluble in water, and benzeneselenol in water at 50–55°. In order to achieve the desired compounds, β-CD was used as a catalyst, and the reactions took place efficiently with good yields (68–88%), as summarized in Table 14. It is worth noting that only the (*Z*)-isomer was obtained when the *Baylis–Hillman* acetates presented an ester moiety in their structure, whilst the nitrile-containing one rendered the (*E*)-isomer as the main trisubstituted alkene (**94** in Table 14) [77].

## 6. Conclusions and Future Prospects

CDs are well-known cyclic oligosaccharides that can enhance the aqueous solubility and stability of a wide range of organic molecules and drugs due to their unique structural characteristics. In aqueous conditions, CDs can be easily biodegraded, as they present a hydrophilic outer side, whereas the inner cavity is hydrophobic and can encapsulate a vast variety of compounds. Despite the overwhelming number of studies related to them, the literature on their application with Se and Te remains limited. Herein, we have compiled all the advances made in this research field, and several conclusions can be drawn:

1. The formation of dimeric CDs through a diselenide or ditelluride bridge can render outstanding structures with protective effects toward several radical species, including ROOH (i.e., H_2_O_2_, CHP, and BHP) and ROS, as many of them have been demonstrated to exert a GPx-mimetic activity. Of note, it has been reported that a great number of these bridged bis-CDs have a greater protective potential than ebselen, a well-studied organoselenium compound. In view of the former, both Se- and Te-bridged CDs can be considered a promising strategy for the treatment of diseases caused by free radicals. Furthermore, some of these CD-based mimics of GPx can provide protection from UV-B radiation. Thus, 2-SeCD was demonstrated to protect NIHT3 fibroblast cells from the oxidative stress generated by this radiation. Likewise, 6-CySeCD was able to impede the apoptosis in the HaCaT cells damaged by UV-B radiation.
2. Among bridged bis-CDs, ditelluride bridges exhibit stronger protective effects than their Se counterparts. For example, 2-TeCD exhibited a GPx-mimicking activity of 34.4 U/µmol, whereas the reported activity for 2-SeCD was 7.4 U/µmol. Additionally, joining two CDs through a SeCys molecule (DisecysCD) led to a higher activity than the one reported for this amino acid, the native β-CD itself, and the combination of both.
3. It appears that the use of different CDs to form guest–host complexes with selenocompounds can present several advantages for the derivatives that exhibit several limitations, i.e., solubility problems and/or volatile nature. This is due to the fact that CDs can modify and improve pivotal properties of these compounds, such as volatility, solubility, degradability, and bioavailability. Therefore, the vehiculation of these compounds could pave the way for using them in a safe and effective way for the treatment of a myriad of disorders. In fact, EβpolySol can be considered a promising candidate for the PrEP treatment in HIV infections, as it has been proven to exert an in vitro effective anti-HIV activity and reduce the vaginal fungal burden and exhibits low toxicity towards cervical cells.
4. A more recent application of CDs in the development of novel Se and Te compounds is their use as catalysts. Thereby, not only can greener conditions be used but much greater yields can be achieved with almost no byproduct formation. This strategy has been demonstrated to be effective in an ample range of reactions, such as cyclizations, oxirane ring opening, and nucleophilic additions to *Baylis–Hillman* acetates. Some reactions can be achieved regio- or stereoselectively.

Although great and interesting achievements have been made in this research field, there are still some aspects that should be taken into account as future perspectives:

1. Further research should prioritize in vivo studies to verify the safety and effectiveness of the described Se- and Te-CDs. This is crucial given that many chalcogen-containing compounds face limitations, such as systemic toxicity, which impede their progression in drug development. On the other hand, data from several in vivo studies support that the formation of complexes with CDs can provide considerable improvements in the oral permeability, pharmacokinetics, stability, solubility, efficacy, and bioavailability of a vast variety of drugs and biologically active molecules such as docetaxel [78], curcumin and its derivatives [79,80], glipizide [81], naproxen [82,83], quercetin [84,85,86,87], tamoxifen [88], galangin [89], agomelatine [90], shikonin [91], resveratrol [92], and hydrochlorothiazide [93]. Therefore, the CD formulation of Se and Te derivatives could be a feasible and promising strategy to surmount their drawbacks, thus improving their clinical relevance.
2. Considering that the use of β-CDs as catalysts in organic reactions can be considered a safe, highly efficient, eco-friendly, and cost-effective strategy, we consider that it would be interesting to further explore the synthesis of a wider range of Se compounds with different functional groups and scaffolds. In this line, it should be highlighted that the synthesis of a wider variety and larger libraries of organotellurium compounds remains a considerable, but worthwhile, challenge, as they usually display unusual reactivities. Albeit there are no reports of the synthesis of Te derivatives catalyzed by CDs, this strategy could nudge the research focus into using greener conditions for obtaining these compounds.
3. CD-based nanosponges (CD-NSs), nanoparticles, and supramolecular hydrogels have attracted great attention in the past years, as CDs have led to several fascinating applications of these materials [94,95,96]. Therefore, studying the role of Se- and Te-CDs could pave the way for discovering novel materials with different potential applications.

To conclude, the use of CDs would have an outstanding impact on the further development of Se and Te research since these macromolecules can ameliorate the main drawbacks of the Se and Te compounds, i.e., poor water solubility and biodistribution profile, stability issues, and toxicity. Furthermore, CDs can also render more complex, active, and selective Se and Te derivatives through greener reaction conditions. Additionally, both chalcogen elements can impact the clinical application of CDs, giving them a plethora of potent bioactivities, including antitumor and antiparasitic ones, among many others. Thus, we consider that CDs and Se and Te could establish a symbiotic relationship.

## Figures and Tables

**Table 3:** Initial rate of reaction (υ_0_) and thiol peroxidase activities of PhSeSePh, PhTeTePh, the native β-CD, and 6-SeCD (**18**) [39].

		υ_0_ (M min^−1^)	Activity	
Ref.	Hydroperoxide	ArSH	ArSH	GSH
PhSeSePh	H_2_O_2_	(0.12 ± 0.01) × 10^7^	1	1
PhTeTePh	H_2_O_2_	(8.03 ± 0.17) × 10^7^	67	0.90
β-CD	H_2_O_2_	0	0	0.00052
**18**	H_2_O_2_	(0.49 ± 0.03) × 10^6^	4083	2.03
	BHP	(3.66 ± 0.12) × 10^6^	30,500	4.12
	CHP	(1.50 ± 0.04) × 10^5^	125,000	8.97

**Table 4:** The GPx-like activity of ebselen and compounds **17** and **19**–**24** [40].

	GPx-Mimicking Activities (U/µmol)		
Ref.	H_2_O_2_	BHP	CHP
Ebselen	0.99 ± 0.01	0.34 ± 0.01	1.24 ± 0.01
**17**	76.71 ± 0.36	104.5 ± 0.73	143.4 ± 0.82
**19**	12.21 ± 0.11	14.97 ± 0.17	18.32 ± 0.51
**20**	7.83 ± 0.25	9.82 ± 0.26	12.33 ± 0.33
**21**	6.08 ± 0.16	7.39 ± 0.08	8.63 ± 0.09
**22**	145.8 ± 0.71	645.3 ± 1.15	832.2 ± 2.56
**23**	22.57 ± 0.40	12.88 ± 0.21	38.51 ± 0.35
**24**	7.23 ± 0.16	3.76 ± 0.08	10.28 ± 0.10

**Table 5:** SOD activities of the native β-CD, oligoamino-modified β-CDs, ebselen, and compounds **25**–**30** [45].

Ref.	Activity (U/mg)	Activity (U/µmol)
β-CD	0	0
β-CDen	0	0
β-CDdien	0	0
β-CDtrien	0	0
β-CDprn	0	0
**25**	179	269
**26**	330	510
**27**	221	343
**28**	206	301
**29**	121	184
**30**	162	262

**Table 6:** GPx-like activities of the native β-CD, oligoamino-modified β-CDs, and compounds **25**–**30** [45].

Ref.	Activity (U/mg)	Activity (U/µmol)
β-CD	361	99
β-CDen	0	0
β-CDdien	0	0
β-CDtrien	0	0
β-CDprn	0	0
Ebselen	3.61	0.99
**25**	0	0
**26**	25	37
**27**	28	43
**28**	31	48
**29**	26	38
**30**	22	34

**Table 7:** Antioxidant activities, Trx and TrxR inhibitory effects, and cancer cell growth inhibiting properties of 6-SeCD (**1**) and compounds **36**–**40** [47].

	GPx-Mimicking Activities (%)			IC_50_ (µM)		
Ref.	H_2_O_2_	BHP	CHP	TxR/Trx	TxR	MCF-7
**1**	100	120	105	18.0	17.8	10.2
**36**	170	560	2075	7.6	1.6	10.1
**37**	230	640	2200	7.6	1.6	4.6
**38**	225	940	1850	4.0	18.0	1.6
**39**	430	1240	2370	3.4	0.26	10.4
**40**	520	3680	6240	1.2	18.0	6.0

**Table 8:** Water solubility of the selenocompounds (**42**–**51**) alone and with β- or γ-CD [51].

	Water Solubility (M)		
Ref.	Compound	Compound + β-CD	Compound + γ-CD
**42**	9.70 × 10^−6^	9.92 × 10^−4^	2.73 × 10^−4^
**43**	2.24 × 10^−5^	5.65 × 10^−5^	5.24 × 10^−5^
**44**	1.20 × 10^−5^	1.57 × 10^−4^	4.39 × 10^−5^
**45**	2.00 × 10^−5^	4.53 × 10^−5^	3.37 × 10^−5^
**46**	5.72 × 10^−6^	4.55 × 10^−5^	4.21 × 10^−5^
**47**	8.43 × 10^−6^	1.01 × 10^−4^	1.51 × 10^−5^
**48**	1.32 × 10^−7^	7.96 × 10^−6^	7.60 × 10^−6^
**49**	6.05 × 10^−7^	2.19 × 10^−7^	6.14 × 10^−6^
**50**	5.57 × 10^−6^	2.70 × 10^−5^	1.77 × 10^−5^
**51**	2.12 × 10^−5^	5.42 × 10^−3^	7.50 × 10^−3^

**Table 9:** In vitro antifungal results of EβpolySol, free ebselen, and two reference drugs (fluconazole and miconazole) [60].

	MIC (µM)	
Ref.	*Candida albicans*	*Candida glabrata*
EβpolySol	20 µM	20 µM
Ebselen (in DMSO)	20 µM	20 µM
Blank film	>500 µM	>500 µM
Fluconazole	>500 µM	>500 µM
Miconazole	100 µM	100 µM

**Table 10:** Compounds obtained following Scheme 1 and their corresponding times of reaction and yields [73].

Ref.	R	X	Time (min)	Yield (%)
**52**	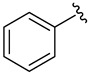	H	40	92
**53**	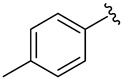	H	45	94
**54**	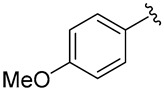	H	40	95
**55**	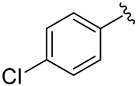	H	50	93
**56**	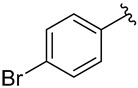	H	50	92
**57**	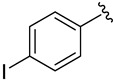	H	50	90
**58**	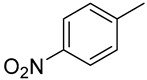	H	35	94
**59**	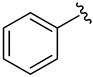	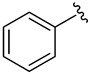	45	91
**60**	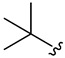	H	60	86
**61**	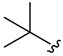	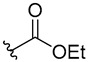	55	87

**Table 11:** Compounds obtained following Scheme 2 and Scheme 3 and their corresponding yields [74].

Ref.	X	Y	R_1_	R_2_	Yield (%)
**62**	H	C	H	H	69
**63**	F	C	H	H	68
**64**	H	C	CH_3_	CH_3_	70
**65**	H	N	CH_3_	CH_3_	72
**66**	CN	C	CH_3_	CH_3_	61
**67**	-	-	-	-	64

**Table 12:** Compounds obtained following Scheme 4 and their corresponding times of reaction and yields [75].

Ref.	R	Time (min)	Yield (%)
**68**	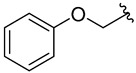	30	86
**69**	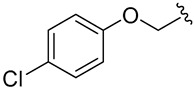	30	86
**70**	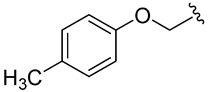	30	84
**71**	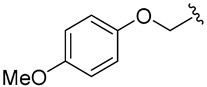	35	82
**72**	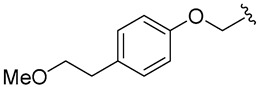	35	82
**73**	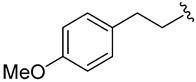	35	80
**74**	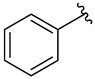	25	78
**75**	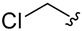	40	75
**76**	-	40	80
**77**	-	40	80

**Table 13:** Compounds obtained following Scheme 5 and their corresponding times of reaction and yields [76].

Ref.	X	R	Time (min)	Yield (%)
**78**	COCH_3_	H	20	88
**79**	CN	H	25	86
**80**	CHO	H	20	85
**81**	CO_2_Me	H	25	86
**82**	CO_2_Me	CH_3_	45	82
**83**	CO_2_Et	H	25	86
**84**	CO_2_Et	CH_3_	45	80
**85**	CONH_2_	H	30	80
**86**	-	-	45	82
**87**	-	-	45	80

**Table 14:** Compounds obtained following Scheme 6 and their corresponding times of reaction and yields [77].

Ref.	R_1_	R_2_	Isomer	Time (h)	Yield (%)
**88**	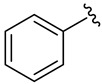	COOMe	*Z*	5	88
**89**	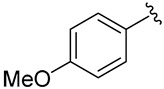	COOEt	*Z*	5	85
**90**	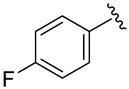	COOEt	*Z*	5	82
**91**	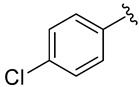	COOEt	*Z*	5	81
**92**	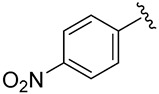	COOMe	*Z*	55	68
**93**	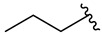	COOEt	*Z*	6	70
**94**	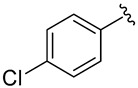	CN	*E*	5	75
